# Supplementary figures and images for: Dickkopf-1 Promotes Angiogenesis and is a Biomarker for Hepatic Stem Cell-like Hepatocellular Carcinoma
Source: Int J Mol Sci. 2022 Mar 3;23(5):2801. doi: 10.3390/ijms23052801 (PMC8911428; doi:10.3390/ijms23052801)

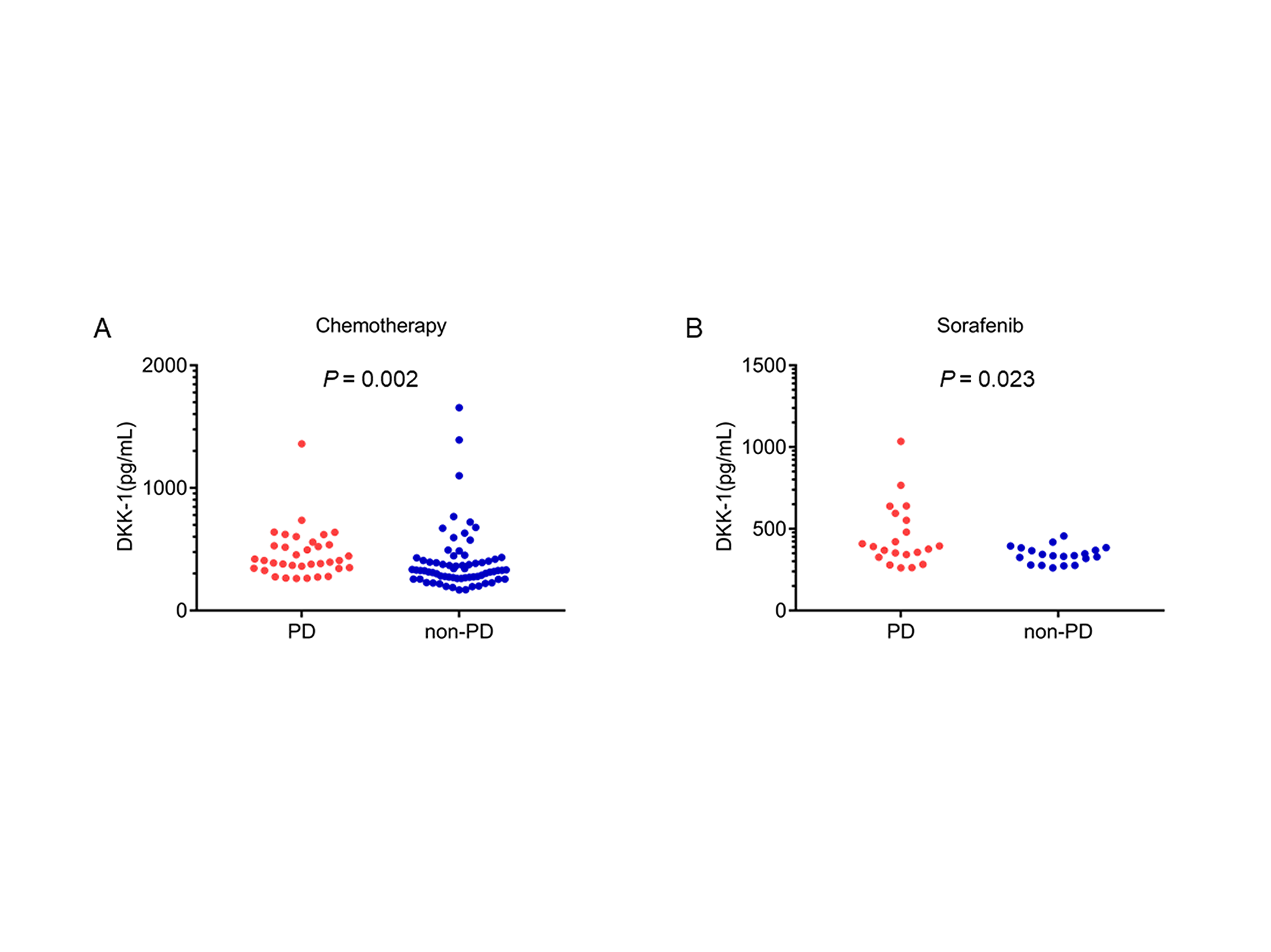

Supplement: Supplementary file 1 [file ijms-23-02801-s001.zip › Supplementary Fig.tif]
